# Presence of TMD-related pain and symptoms associated with anxiety in Peruvian students in their final years of dental education: an analytical cross-sectional study under a multivariable regression model

**DOI:** 10.1186/s12903-025-05638-7

**Published:** 2025-02-21

**Authors:** Karen Angeles-García, Marysela Ladera-Castañeda, Leonor Castro-Ramirez, Elizabeth Paucar-Rodríguez, Miriam Castro-Rojas, Luis Cervantes-Ganoza, César Cayo-Rojas

**Affiliations:** 1 Postgraduate School, Universidad Nacional Federico Villarreal, Research Team “Salud Pública – Salud Integral”, Lima, Peru; 2 School of Stomatology, Universidad Privada San Juan Bautista, Lima, Peru; 3 Faculty of Stomatology, Universidad Inca Garcilaso de la Vega, Lima, Peru

**Keywords:** Temporomandibular disorders, Anxiety, Dental students, Dental education

## Abstract

**Background:**

Temporomandibular disorders (TMD) are frequently associated with anxiety, as this can increase the hyperactivity of the masticatory muscles, resulting in TMD-related pain and symptoms. The aim of this study was to assess the presence of TMD-related pain and symptoms associated with anxiety levels in Peruvian students in their final years of dental education.

**Methods:**

This analytical cross-sectional study of 273 Peruvian students in the final two years of dental education was conducted from October to December 2023. The Zung test was employed to diagnose anxiety, while the TMD-Pain Screener questionnaire was utilized to diagnose painful temporomandibular disorders (TMD). A Poisson regression model with robust variance using Adjusted Prevalence Ratio (APR) was employed to assess the prevalence of TMD-related pain and symptoms. The following variables were considered in the analysis: anxiety, sex, age, academic year, marital status, area of residence, type of housing, living with parents, and occupation. All statistical tests were conducted with a significance level of *p* < 0.05.

**Results:**

The prevalence of TMD-related pain and symptoms was 24.5%. The 0.7% of the sample exhibited very extreme levels of anxiety; 8.1% demonstrated severe anxiety; and 39.9% exhibited mild to moderate anxiety. Furthermore, dental students with severe to very extreme anxiety and with mild to moderate anxiety were 8.2 times and 3.8 times, respectively, more likely to present TMD-related pain and symptoms (APR = 8.18, 95% CI: 4.62–14.47 and APR = 3.84, 95% CI: 2.18–6.75, respectively), compared to those who did not have anxiety. Conversely, no significant association was observed between the presence of TMD-related pain and symptoms and sex, age, academic year, marital status, area of residence, type of housing, living with parents, or occupation (*p* > 0.05).

**Conclusion:**

Almost a quarter of the students in their final years of dental education had TMD-related pain and symptoms. It was found that as the level of anxiety increased, from mild to moderate and from severe to very extreme, the likelihood of experiencing TMD-related pain and symptoms also increased significantly. On the other hand, gender, age, academic year, marital status, area of residence, type of housing, living with parents or occupation were not found to be influential factors in the presence of TMD-related pain and symptoms.

**Supplementary Information:**

The online version contains supplementary material available at 10.1186/s12903-025-05638-7.

## Background

Temporomandibular disorders (TMD) are musculoskeletal and neuromuscular conditions that can affect the temporomandibular joint (TMJ), masticatory muscles, and/or their associated structures [1–3]. It is estimated that approximately 60–70% of the general population will experience at least one symptom of TMD at some point in their lives [4–9]. Some studies have indicated that this disorder may affect 5–12% of the population [6, 8], while others have reported incidences of 31% [10] and 77% [11]. With regard to gender, the available literature indicates that the occurrence is predominantly female [6, 8, 9, 11]. Anxiety is one of the most frequently identified risk factors for TMDs [10–12]. In addition, it can modify pain sensations and release neurotransmitters related to parafunctional habits, which can further aggravate the condition. In addition, it can intensify the hyperactivity of the masticatory muscles associated with the TMJ, thereby increasing the risk of joint overload and accelerating the onset of TMDs [1, 13, 14].

Weitzman et al. [15] have previously highlighted the significance of universities as a setting for the study of mental health in young people. They have observed that the pursuit of a university degree often provokes a high degree of anxiety in students [14, 16]. Furthermore, students are confronted with higher societal expectations in increasingly competitive environments compared to previous generations [17, 18]. Given the relationship between anxiety and TMD, it could be presumed that university students are particularly susceptible to developing this disorder [13, 14]. Some reports indicate that anxiety most commonly affects 18 to 25 year [19–21]. In addition, it has been documented that dental students are particularly prone to develop anxiety from the onset of the pandemic [22–24]. This is even more so if they are in the final years of their dental training, undertaking an internship in the university clinic or in a hospital, given the potential risk of contracting a new mutation of the coronavirus through contact with infected patients [24] or perhaps concerns about contracting new infectious diseases.

The TMD-Pain Screener is a component of the DC/TMD questionnaire and is employed to assess the presence of orofacial pain in a range of daily situations that are associated with TMDs [25–28]. This instrument assesses pain, jaw stiffness and factors that may affect pain, such as function and parafunction. The rationale for the use of this questionnaire is based on its reliability and validity measures. The estimates have an internal reliability level ranging from 0.87 to 0.93, with sensitivity values of 99% and specificity of 97% [29]. In addition, an acceptable internal consistency of the TMD-Pain Screener questionnaire was verified in a sample of 2562 dental students in Peru, from 21 different geographical locations, including different regions such as the Peruvian coast, the highlands and the Amazon jungle [26]. It is crucial to determine whether the anxiety observed in some dental students in the post-pandemic era is causally related to TMDs. Establishing such an association would allow dental professionals to implement effective interventions to mitigate the occurrence of TMDs in this population, in collaboration with behavioral science professionals. In addition, early detection of TMDs through symptoms, as well as identification of the factors associated with them, will contribute to taking timely measures to reduce their impact on quality of life [30].

The aim of this study was to assess the presence of TMD-related pain and symptoms associated with anxiety levels in Peruvian students in their final years of dental education. The null hypothesis was that the presence of TMD-related pain and symptoms is not significantly associated with anxiety levels in Peruvian students in their final years of dental education.

## Methods

### Type of study and delimitation

This analytical cross-sectional study was written in accordance with the Strengthening the Reporting of Observational Studies in Epidemiology (STROBE) guidelines for observational studies [31]. The study was conducted between October and December 2023 at the Universidad Católica Los Ángeles de Chimbote, which is headquartered in the province of Chimbote, Peru and has a branch in the province of Trujillo, Peru.

### Population and selection of participants

**Fig. 1:**
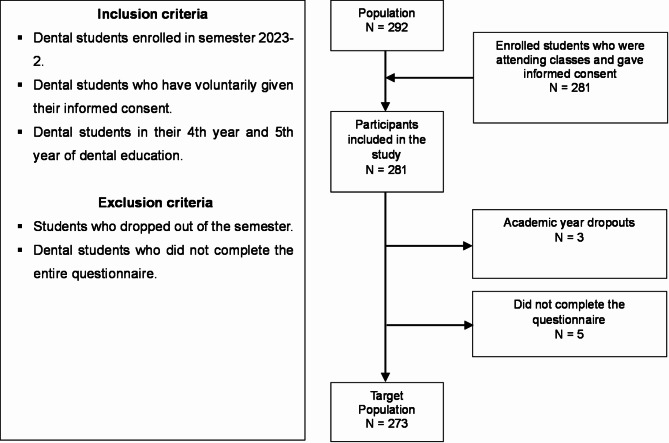
Flowchart on participant selection

The total population consisted of 292 dental students: 113 from Trujillo and 179 from Chimbote. A total of 187 fourth-year students and 105 fifth-year students were enrolled. The minimum sample size according to the association of Anxiety versus TMD-related pain and symptoms in a previous analysis with 50 participants was *n* = 70 in the statistical package G*power 3.1.9.7, considering a significance level α = 0.05, a statistical power (1 - β) = 0.80, an effect size = 0.34 and degree of freedom = 1. It was decided to include the entire study population while respecting the eligibility criteria. Inclusion criteria were male and female students enrolled in semester 2023-2, who signed the informed consent form and were in their 4th and 5th year of dental education. Students who dropped out of the academic semester were excluded and those who did not complete the questionnaire were also excluded. Consequently, the final target population was *N* = 273, comprising 179 fourth-year students and 94 fifth-year students [Fig. 1]:

### Variables

The dependent variable considered in this study was TMD-related pain and symptoms, and the independent variable was anxiety. Possible confounding variables were sex [22, 24], age group [22, 24], academic year [22], marital status [22, 24], area of residence [24], type of housing, occupation [24], and living with parents.

### Application of the instrument

The Zung Self-Assessment Anxiety Scale (SAS), previously validated in Peruvian dental students [21], was employed to identify anxiety symptoms [See supplementary material]. For TMD-related pain and symptoms, the TMD-Pain Screener questionnaire was employed [25–28], which had previously been validated in Peruvian dental students [26] [See supplementary material]. The internal consistency of both tests was optimal, with a Cronbach’s alpha (α) of 0.84 (95% CI: 0.82–0.87) for the first test and 0.75 (95% CI: 0.70–0.79) for the second [19].

The SAS scale comprised 20 items distributed across two dimensions. The first dimension consisted of five items pertaining to psychological or affective signs and symptoms, while the second dimension consisted of 15 items pertaining to somatic signs and symptoms. The questions were directed towards the students’ perceptions of their experiences over the 15 days prior to the evaluation. Each item was answered on a four-point Likert scale. The response options were “never or rarely” (1 point), “sometimes” (2 points), “a good number of times” (3 points), and “most of the time” (4 points). The total score was converted into an Anxiety Self-Assessment Scale Index (SAS Index), which was calculated as follows: The total score was divided by 80 and multiplied by 100. The final diagnosis was based on the following categories: absent (less than 45), mild to moderate (45 to 59), severe (60 to 74), and very extreme (75 or more) [21].

The TMD-Pain Screener questionnaire consisted of three questions, with the first one having three alternatives, namely “a,” “b,” and “c.” The other questions had two alternatives, namely “a” and “b.” Only the third question included four items (A, B, C, and D). A score of zero was assigned to a response of “a,” one point to a response of “b,” and two points to a response of “c.” The absence of TMD-related pain and symptoms was considered to be from 0 to 2 points, while the presence of TMD-related pain and symptoms was considered to be from 3 points or more [26].

### Procedure

The questionnaires were distributed at the end of the students’ regular classes between October 10 and December 20, 2023. The heteroadministered survey was conducted by the principal investigator. (KAG). Before the students answered the questionnaires, the researcher provided them with informed consent that included the purpose of the study, the institutional email address, the phone number, and the full name of the principal investigator. The details of the institutional ethics committee that approved the study were also communicated. Students who voluntarily gave their informed consent were provided with the questionnaire and instructions for completing it. The principal author who administered the questionnaires worked at the institution where the students were surveyed. However, to avoid information bias, the survey was shared with them without direct supervision, and they were given a reasonable amount of time to fill out the questionnaires in the classroom, while others preferred to participate outside of it. Moreover, the principal investigator did not hold any position of authority that would have induced the students to feel pressured to participate. They were also not asked for personal information such as name or phone number. Additionally, the participants were informed that they had the full right to decline the invitation or not complete the questionnaire if they wished. The principal investigator tabulated the data, and subsequently, only the researchers had access to the data, which were coded with their initials and age. (e.g., KAG45). Additionally, the data was password-protected on a portable digital device to ensure the confidentiality of the information. The students participated in the study only once and did not receive any compensation for their participation. The results were communicated to everyone who requested them via email to the principal investigator.

### Data analysis

The data were processed and analyzed using the Statistical Package for the Social Sciences (SPSS) version 28.0. For the descriptive analysis of quantitative variables, such as age, the mean, median, and standard deviation were employed. For qualitative variables, absolute and relative frequencies were employed, along with a bar graph. A Poisson regression model with robust variance using Adjusted Prevalence Ratio (APR) was employed for multivariable analysis. All statistical tests were conducted with a significance level of *p* < 0.05.

### Ethics issues

This study adhered to the ethical principles set forth in the Declaration of Helsinki, which emphasize respect, freedom, nonmaleficence, and confidentiality [32]. Furthermore, the study was reviewed and approved by the Ethics Committee of the Graduate School of the Universidad Nacional Federico Villarreal, with approval of Act No. 020-2023-UIIE-EUPG-UNFV dated October 2, 2023. Prior to commencing the questionnaire, participants were requested to provide their voluntary informed consent.

## Results

The mean age of the dental students was 25.1 ± 4.2 years, with 57.1% of the cohort being younger than 25 years. The total number of participants was 61.5% female and 65.6% in their fourth year of studies. Furthermore, 84.2% of the participants were single, and 82.4% resided in urban areas. Additionally, 77.7% of the total population resided in their own domicile, while 46.9% lived with both parents. Finally, 65.9% of the participants reported that they were only studying [Table 1].

**Table 1:** Sociodemographic characteristics of dental students at a private university

Variable	Category	Frequency	Percentage
**Age group**	< 25 years	156	57.1
	≥ 25 years	117	42.9
**Sex**	Male	105	38.5
	Female	168	61.5
**Academic year**	4th year	179	65.6
	5th year	94	34.4
**Marital status**	Single	230	84.2
	Married or cohabiting	43	15.8
**Area of residence**	Urban	225	82.4
	Rural	48	17.6
**Type of housing**	Owned	212	77.7
	Rented	61	22.3
**Living with parents**	Both parents	128	46.9
	Mother only	50	18.3
	Other family member	49	17.9
	Alone	46	16.8
**Occupation**	Just studying	180	65.9
	Studying and working	93	34.1
**Age**	**Mean**	**Median**	**SD**
	25.1	24.0	4.2

SD: Standard deviation

**Fig. 2:**
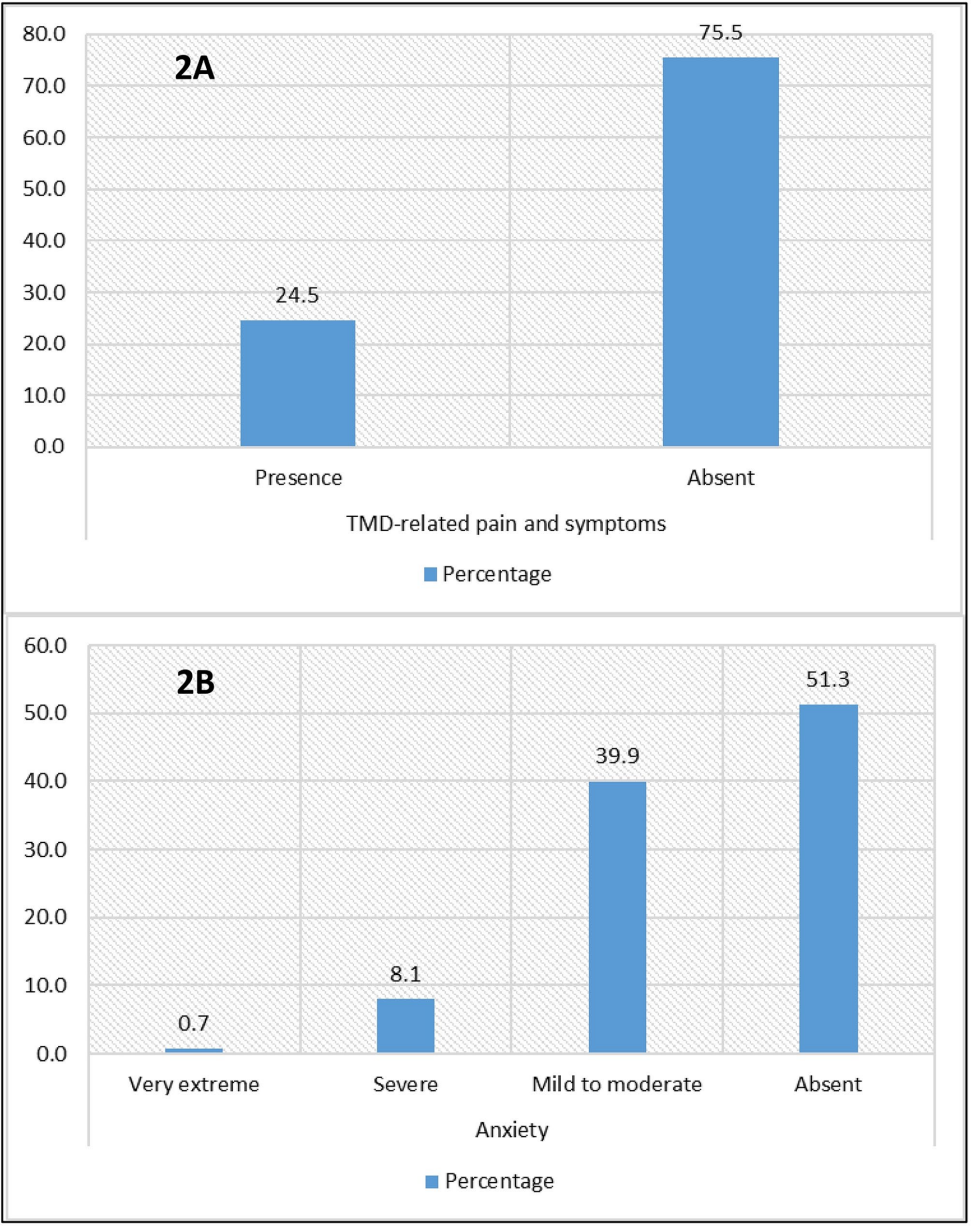
**2A:** Frequency of TMD-related pain and symptoms. **2B**: Anxiety levels in students in their final years of dental education

A total of 24.5% (95% CI: 19.4–29.6%) of the 273 dental students exhibited TMD-related pain and symptoms. Furthermore, 0.7% (95% CI: 0.0–1.7%) exhibited very extreme anxiety, 8.1% (95% CI: 4.8–11.3%) demonstrated severe anxiety, and 39.9% (95% CI: 34.1–45.7%) exhibited mild to moderate anxiety [Fig. 2A y 2B].

In the crude model of the simple Poisson regression analysis with robust variance using the prevalence ratio, the dependent variable was considered to be TMD-related pain and symptoms (presence = 1, absence = 0), while the independent variable was anxiety levels. In addition, age group, sex, year of study, marital status, residence, type of housing, living with parents, and occupation were considered as possible confounding variables. When adjusting the prevalence ratio of the Poisson multiple regression model with robust variance, including all categories of the independent variable with *p* < 0.05 analysed from the crude model, it was observed that dental students with severe to very extreme anxiety and mild to moderate anxiety were 8.2 times and 3.8 times more likely, respectively, to present TMD-related pain and symptoms (APR = 8.18, 95% CI: 4.62–14.47 and APR = 3.84, 95% CI: 2.18–6.75) compared to those who did not have anxiety. Furthermore, it was found that none of the confounding variables considered in this study had a significant influence on the presence of TMD-related pain and symptoms (*p* > 0.05) [Table 2].

**Table 2:** Regression analysis model of TMD-related pain and symptoms associated with anxiety levels and possible confounding variables

Variable	Category	Crude model						Adjusted model				
		β	PR	95% CI		p*		β	APR	95% CI		p**
				LL	UL					LL	UL	
**Anxiety**	Severe to very extreme	1.96	7.08	4.05	12.39	< 0.001*		2.10	8.18	4.62	14.47	< 0.001**
	Mild to moderate	1.19	3.30	1.88	5.81	< 0.001*		1.35	3.84	2.18	6.75	< 0.001**
	Absent		*Ref.*						*Ref.*			
**Age group**	< 25 years	0.04	1.04	0.68	1.59	0.839		0.08	1.09	0.70	1.68	0.710
	≥ 25 years		*Ref.*						*Ref.*			
**Sex**	Male	-0.25	0.78	0.50	1.22	0.282		0.24	1.27	0.83	1.96	0.275
	Female		*Ref.*						*Ref.*			
**Year of study**	4th year	-0.06	0.94	0.61	1.45	0.782		-0.03	0.97	0.63	1.49	0.894
	5th year		*Ref.*						*Ref.*			
**Marital status**	Single	-0.05	0.95	0.54	1.66	0.862		-0.34	0.71	0.39	1.28	0.257
	Married or cohabiting		*Ref.*						*Ref.*			
**Area of residence**	Urban	0.08	1.09	0.62	1.91	0.775		0.05	1.06	0.60	1.87	0.854
	Rural		*Ref.*						*Ref.*			
**Type of housing**	Owned	-0.09	0.92	0.57	1.49	0.726		0.02	1.02	0.63	1.65	0.942
	Rented		*Ref.*						*Ref.*			
**Living with parents**	Both parents	0.45	1.57	0.79	3.14	0.199		0.41	1.50	0.75	3.01	0.248
	Mother only	0.14	1.15	0.50	2.66	0.744		0.15	1.16	0.55	2.47	0.696
	Other family member	0.50	1.64	0.76	3.55	0.206		0.69	1.99	1.00	3.97	0.051
	Alone		*Ref.*						*Ref.*			
**Occupation**	Only studying	-0.08	0.93	0.60	1.43	0.726		-0.17	0.85	0.55	1.31	0.453
	Studying and working		*Ref.*						*Ref.*			
*Model constant*								-2.54	0.08	0.03	0.24	< 0.001

*Simple Poisson regression model with robust variance (*p < 0.05, significant association), PR: crude prevalence ratio. **Adjusted model using multiple Poisson regression with robust variance (**p < 0.05, significant association). All categories of the independent variable of the crude model with *p* < 0.05 were included in the adjusted model. APR: Adjusted Prevalence Ratio; β: Coefficient of determination. 95% CI: 95% Confidence Interval; LI: Lower Limit, UL: Upper Limit

## Discussion

Anxiety is a frequently comorbid condition associated with TMD, as it can alter pain sensations and facilitate the release of neurotransmitters related to parafunctional habits [1, 3, 14]. The objective of this study was to assess the presence of TMD-related pain and symptoms associated with anxiety levels in Peruvian students in their final years of dental education. With the results obtained, the null hypothesis was rejected.

This study found that the prevalence of TMD-related pain and symptoms was 24.5%, which is slightly higher than that found by Chuinsiri et al. [28], who reported a prevalence of 22.2% in Thailand. In contrast, Alrashdan et al. [33] reported a prevalence of 20.7% in Jordan, and Jeremic-Knezevic et al. [34] reported a prevalence of 16.4% in Serbia. These discrepancies may be attributed to the distinctive attributes of the studied populations. Previous research has documented cross-cultural variations in pain perception, with African Americans exhibiting heightened sensitivity to pain compared to non-Hispanic whites, non-white ethnic groups, and Hispanic individuals of mixed ancestry [35, 36]. Given that Peru is a multicultural, pluriethnic, and megadiverse country [33, 37], it is possible that the intercultural characteristics of the population may have influenced the observed differences in the results.

In this study, 0.7% of dental students exhibited very extreme anxiety, 8.1% demonstrated severe anxiety, and 39.9% exhibited mild to moderate anxiety. Studies such as those conducted by Salah El-Din et al. [23], Homeida et al. [38], and Castro et al. [24] have reported higher anxiety values. This discrepancy could be attributed to the use of instruments such as the Generalized Anxiety Disorder 7-item (GAD-7) [23], the Patient Health Questionnaire-4 PHQ (PHQ-4) [38], and the Depression, Anxiety, and Stress Scale-21 items (DASS-21) [24]. Furthermore, the discrepancy in results may be attributed to the fact that the study was conducted during the early stages of the pandemic, when there was a heightened risk of infection by the novel coronavirus and mandatory social isolation had been mandated [39]. It is important to note that the sensitivity and specificity estimates of different anxiety scales may vary due to the cut-off points used to diagnose anxiety. Consequently, the Zung scale has been identified as the most stable, given that the cut-off score remains relatively consistent [40, 41]. Nevertheless, all studies demonstrated a positive association between TMD and anxiety.

The results of this study indicate that dental students with severe to very extreme anxiety and mild to moderate anxiety were 8.2 times and 3.8 times, respectively, more likely to have TMD-related pain and symptoms compared to those without anxiety. This finding is consistent with previous research by Homeida et al. [36] and Chuinsiri et al. [28], which demonstrated that anxiety levels were associated with TMD and that participants diagnosed with painful TMD demonstrated substantially higher anxiety. This is likely due to the fact that anxiety increases the activity of the masticatory muscles, causes constant pressure on the teeth, and alters local circulation in the muscles and ionic exchange between cell membranes. Additionally, it can result in the accumulation of lactic acid and pyruvic acid, which can trigger the release of neurotransmitters that could stimulate pain receptors in the joints [1, 3, 14, 42, 43].

The present study revealed that age, sex, academic year, marital status, area of residence, type of housing, cohabitation with parents, and dedication were found to be significant factors influencing the presence of TMD-related pain and symptoms. Notably, no association was found between gender and the presence of TMD-related pain and symptoms, which was inconsistent with several previous studies [2, 4, 6, 13, 28, 33, 44]. This may be attributed to physiological characteristics, particularly hormonal variations and differences in connective tissue and muscle laxity, which are related to estrogen levels. This may in turn influence the different sex-specific functional pressure responses, which may lead to TMD-related pain and symptoms [2, 13, 28, 33, 44]. Furthermore, the intensity of pain has been observed to fluctuate throughout the menstrual cycle in women with TMD. This is attributed to the fact that pain intensity is greater when estrogen concentrations are higher [2, 31, 44, 45]. A number of cell types within the temporomandibular joint (TMJ), including synoviocytes and neurons, express estrogen receptors. It is thought that the activation of these receptors contributes to joint hypermobility, increased matrix metalloproteinase activity, and decreased collagen and protein content in the articular disc. The remaining sociodemographic variables were found to be unrelated to the occurrence of TMD-related pain and symptoms. This could be attributed to the fact that students in their final years of dental education, regardless of their place of residence, age, or living arrangements, exhibited minimal concern or emotional distress. This contrasts with the period of the pandemic, during which the case fatality rate for COVID-19 was significantly higher, particularly among older adults [46, 47].

The questionnaire used to detect anxiety includes both affective and somatic symptoms [41], as anxiety is known to be associated with headaches and back pain [48–50]. Somatization, a common symptom among patients with psychosocial conditions [51], is reflected in question 7 of the SAS. There is substantial evidence that anxiety has a psychosomatic impact, increasing the severity and pain sensitivity of TMD [52–54]. Consequently, chronic pain conditions such as back pain and headaches are prevalent in individuals at risk of developing symptomatic TMD, especially those with a certain psychological profile [10].

This study’s strength lies in its evaluation of anxiety levels in students in their final years of dental education in the context of the post-pandemic era of the novel coronavirus disease (COVID-19). Despite the World Health Organization (WHO) declaring the end of the pandemic on 5 May 2023. The data, collected at the end of 2023, suggest that anxiety remains a significant risk factor for temporomandibular disorders (TMD). However, it is important to note that while anxiety can contribute to TMD, there are also protective factors that mitigate this risk. Relying solely on the TMD Pain Screener to draw conclusions about the causal relationship between pandemic-induced anxiety and TMD-related pain and symptoms should be taken with caution. A comprehensive diagnostic approach is needed to accurately assess the impact of anxiety on the development of TMDs. Another strength of this study is the application of multivariate causality statistical analysis to evaluate possible determinants in the occurrence of TMD-related pain and symptoms.

As a limitation, it should be noted that the sample is not representative of the entire population of Peruvian students in the final years of dental education. However, it constitutes a starting point for developing future epidemiological studies at the local, regional, national, and international levels in this line of research. Furthermore, the cross-sectional design of this investigation did not permit an evaluation of the durability of dental students’ TMD-related pain and symptoms in conjunction with anxiety over time. Longitudinal studies are required to evaluate the long-term impact of anxiety on the development of TMD-related pain and symptoms.

It is recommended that the association between anxiety, bruxism, and TMD be evaluated, as most individuals with anxiety disorders tend to relieve their symptoms by clenching and/or grinding their teeth and contracting the masticatory muscles, which activates the stomatognathic system [23]. Furthermore, it is recommended that external factors such as examinations, clinical requirements, and academic assignments be included in future studies, as these could also trigger some degree of anxiety. Conversely, although the TMD-Pain Screener is a practical tool for TMD-related pain and symptoms, it is recommended that both axes of the DC/TMD questionnaire be applied in future studies, as the TMD-Pain Screener is part of Axis I of the DC/TMD questionnaire. The full protocol is designed for use in any clinical setting and supports the full range of diagnostic activities, from early detection to assessment and definitive diagnosis [25]. Finally, it would be advisable for academic authorities to implement mental health prevention measures in their students or to develop some psychological containment plan in order to mitigate the impact of anxiety on the stomatognathic system [55].

## Conclusion

Almost a quarter of the students in their final years of dental education had TMD-related pain and symptoms. It was found that as the level of anxiety increased, from mild to moderate and from severe to very extreme, the likelihood of experiencing TMD-related pain and symptoms also increased significantly. On the other hand, gender, age, academic year, marital status, area of residence, type of housing, living with parents or occupation were not found to be influential factors in the presence of TMD-related pain and symptoms.

## Electronic supplementary material

Below is the link to the electronic supplementary material.

Supplementary Material 1

## Data Availability

All data analyzed during this study are available from the corresponding author on reasonable request (cesarcayorojas@gmail.com).

## Abbreviations

APR: Adjusted Prevalence Ratio
CI: Confidence interval
COVID-19: COronavirus DIsease 2019
DC/TMD: Diagnostic Criteria for Temporomandibular Disorders
STROBE: STrengthening the Reporting of OBservational studies in Epidemiology
SPSS: Statistical Package for the Social Sciences
TMD: Temporomandibular disorders
