# Supplementary material for: Presence of TMD-related pain and symptoms associated with anxiety in Peruvian students in their final years of dental education: an analytical cross-sectional study under a multivariable regression model
Source: BMC Oral Health. 2025 Feb 21;25:277. doi: 10.1186/s12903-025-05638-7 (PMC11846219; doi:10.1186/s12903-025-05638-7)
Supplement: Supplementary file 1 — Supplementary Material 1 [file 12903_2025_5638_MOESM1_ESM.docx]

**Zung Self-Rating Anxiety Scale (SAS)**

Q1. I feel more nervous and anxious than usual.

Q2. I feel afraid for no reason at all.

Q3. I get upset easily or feel panicky.

Q4. I feel like I’m falling apart and going to pieces.

Q5. I feel that everything is all right and nothing bad will happen.

Q6. My arms and legs shake and tremble.

Q7. I am bothered by headaches, neck and back pains.

Q8. I feel weak and get tired easily.

Q9. I feel calm and can sit still easily.

Q10. I can feel my heart beating fast.

Q11. I am bothered by dizzy spells.

Q12. I have fainting spells or feel faint.

Q13. I can breathe in and out easily.

Q14. I get feelings of numbness and tingling in my fingers and toes.

Q15. I am bothered by stomachache or indigestion.

Q16. I have to empty my bladder often.

Q17. My hands are usually dry and warm.

Q18. My face gets hot and blushes.

Q19. I fall asleep easily and get a good night’s rest.

Q20. I have nightmares.

**TMD-Pain Screener questionnaire.**

1. In the last 30 days, on average, how long did any pain in your jaw or temple area on either side last?

a. No pain

b. From very brief to more than a week, but it does stop

c. Continuous

2. In the last 30 days, have you had pain or stiffness in your jaw on awakening?

a. No

b. Yes

3. In the last 30 days, did the following activities change any pain (that is, make it better or make it worse) in your jaw or temple area on either side?

A. Chewing hard or tough food

a. No

b. Yes

B. Opening your mouth or moving your jaw forward or to the side

a. No

b. Yes

C. Jaw habits such as holding teeth together, clenching, grinding or chewing gum

a. No

b. Yes

D. Other jaw activities such as talking, kissing or yawning

a. No

b. Yes
